# From Laboratory to Field: Unveiling Microbial Community Dynamics to Optimize a Bioaugmentation System with Engineered Redox Zones for Nitrogen Removal in Polluted Waters

**DOI:** 10.3390/microorganisms14081743

**Published:** 2026-08-08

**Authors:** Zifan Li, Mingxian Han, Jingwei Wei, Hongchen Jiang

**Affiliations:** 1 State Key Laboratory of Geomicrobiology and Environmental Changes, China University of Geosciences, Wuhan 430074, China; 1202411498@cug.edu.cn (Z.L.); jingweiwei5525@163.com (J.W.); 2 School of Life Sciences, Henan University, Kaifeng 475004, China

**Keywords:** nitrifying microbial community, aerobic-anoxic process, receiving water, microbial community, in-situ treatment system

## Abstract

The remediation of nitrogen-polluted receiving waters remains a significant challenge, particularly due to the frequent disconnect between understanding the mechanistic action of microbial inoculants and designing effective, field-applicable treatment processes. This study aimed to bridge this gap by evaluating a commercial composite microbial inoculant and translating the insights into a practical in situ system. Laboratory-scale experiments identified an “aerobic-anoxic” operational mode as optimal, achieving an initial NH_4_^+^-N removal efficiency of 95.6% (reducing from 55.6 mg/L to <1.0 mg/L) within the first 7 days. Simultaneously, a distinct three-stage nitrogen transformation process was established, driving the terminal total nitrogen (TN) concentration down to a remarkable 2.6 mg/L, which corresponds to a superior cumulative TN removal efficiency of 95.90%. Concurrently, robust organic matter degradation and phosphorus clearance were achieved, yielding final laboratory removal efficiencies of 67.4% for COD and 6.4% for TP. High-throughput sequencing revealed that this performance was driven by significant microbial succession and environmental filtering, with functional genera such as *Sediminibacterium* (contributing to early-stage organic degradation and nitrogen transformation) and *Kocuria* (acting as metabolic drivers under transitioning redox conditions) becoming predominant during specific degradation phases. Based on these mechanistic findings, a pilot-scale, partitioned treatment system (aerobic bio-contact oxidation zone followed by a hybrid anoxic zone) was designed and implemented in a black-odorous river channel. The system demonstrated robust performance over 60 days, sustaining average removal rates of 93.2% for NH_4_^+^-N and 91.3% for TN. This work provides a validated framework that directly links the elucidation of microbial community dynamics under engineered conditions to the successful development of a manageable bioaugmentation strategy for treating nitrogen-contaminated receiving waters.

## 1. Introduction

The escalating discharge of nitrogenous pollutants into receiving water bodies, stemming from agricultural runoff, industrial effluents, and inadequately treated domestic sewage, constitutes a pervasive threat to aquatic ecosystems globally. Excessive nitrogen loading, primarily in the forms of ammonium (NH_4_^+^-N) and nitrate (NO_3_^−^-N), fuels eutrophication, leads to hypoxia or anoxia, and disrupts biodiversity, posing significant challenges for water quality management and ecosystem sustainability [1,2]. Conventional biological nitrogen removal (BNR) processes, predominantly implemented in centralized wastewater treatment plants (WWTPs), rely on the sequential action of nitrification (aerobic conversion of NH_4_^+^ to NO_3_^−^) and denitrification (anoxic reduction of NO_3_^−^ to N_2_). Exogenous bioaugmentation in open lotic ecosystems is frequently bottlenecked by intense competitive exclusion from indigenous microbiomes and thermodynamic limitations. While effective for point-source pollution, these engineered systems are often ill-suited for the remediation of non-point source pollution in extensive, open receiving waters like rivers, lakes, and coastal zones due to high infrastructure costs, energy consumption for aeration, and the requirement for controlled reactor environments [3,4,5,6]. To overcome these limitations, semi-pilot and field-scale remediation using composite capping agents—such as lanthanum-modified bentonite (Phoslock) and iron-modified bentonite (Bephos)—has gained global attention for proactive nutrient interception and phosphorus binding [7].

In this context, bioaugmentation—the introduction of specific, cultured microorganisms to enhance the degradation of target pollutants—has emerged as a promising in situ bioremediation strategy for such diffuse pollution scenarios [8,9]. The application of microbial inoculants, particularly those containing nitrifying and denitrifying bacteria, offers a potentially cost-effective and flexible approach to accelerate natural attenuation processes in polluted water bodies [10,11]. Recent advancements have shifted from applying single strains to developing complex, synergistic microbial consortia designed for robust environmental performance [1,12,13]. Specifically, recent studies have successfully engineered synthetic functional consortia capable of simultaneous nitrification and denitrification (SND) and heterotrophic nitrification-aerobic denitrification (HN-AD), achieving stable total nitrogen (TN) removal efficiencies exceeding 90% even under severe metabolic shock or high-strength ammonium loads. Furthermore, the integration of newly discovered metabolic pathways—such as completely ammonia-oxidizing (comammox) bacteria and anaerobic ammonium oxidation (anammox) consortia—has demonstrated a remarkable paradigm shift, mitigating nitrite/nitrate accumulation and strengthening community resistance against continuous environmental filtering in open aquatic matrices. Furthermore, studies have demonstrated successful bioaugmentation in various systems, such as enhancing nitrogen removal in subsurface infiltration systems [14] and intensifying biofilm processes for wastewater treatment [10,15]. Specifically, recent field-scale trials successfully accelerated initial ammonium oxidation rates via the continuous dosing of encapsulated consortia.

However, a critical gap persists between promising laboratory-scale results and reliable, effective field-scale application. Many studies on microbial inoculants focus primarily on evaluating removal efficiencies of specific strains or consortia under ideal, controlled laboratory conditions (e.g., synthetic wastewater, constant temperature, optimized C/N ratios) [15,16]. There is a notable scarcity of research that systematically bridges the gap from mechanistic understanding of microbial community dynamics during pollutant removal to the pragmatic design and validation of simple, deployable engineering systems tailored for real-world, flowing water bodies. Despite these advancements, a fundamental knowledge gap remains regarding the ecological persistence and metabolic transitions of bioaugmented consortia under fluctuating environmental gradients. Specifically, current research lacks quantitative insights into how introduced functional strains adapt to organic-rich shocks, how native communities respond to foreign inoculation, and how sequential redox transitions govern the metabolic relay between nitritation, nitratation, and heterotrophic denitrification. Addressing these factual uncertainties is crucial for transforming empirical bioaugmentation into a controllable, field-applicable engineering technology. While site-specific isolation yields highly adapted consortia, utilizing a standardized commercial inoculant allows for broader replication and scaling. This choice serves our primary objective: to prove that engineering custom redox niches can successfully unlock the performance of mass-produced commercial bioagents, which typically face field failures due to poor ecological integration.

Addressing these questions is essential for advancing bioaugmentation from a laboratory concept to a dependable field technology. Recent work has begun to explore these interfaces. For example, research on dirammox-dominated communities highlights the potential of alternative nitrogen pathways [17], while other studies delve into microbial community dynamics during process upgrades in full-scale plants [18] or within integrated aquaculture systems [19]. The design of novel anaerobic/aerobic/anoxic (AOA) processes also reflects the ongoing pursuit of efficient, carbon-saving nitrogen removal configurations [4,20]. Yet, an integrated study that cohesively links inoculant performance assessment, microbial mechanism elucidation under designed redox zones, and subsequent field demonstration via a custom-built, simple reactor remains lacking.

This study therefore posits the following central hypothesis: A composite nitrifying microbial inoculant, when applied within a precisely pre-designed “aerobic-anoxic” partitioned treatment process, can achieve efficient and stable nitrogen removal in receiving water bodies by fostering a synergistic microbial community that leverages sequential nitrification and denitrification pathways, and this integrated bioaugmentation-engineering approach can be effectively demonstrated at pilot scale. To test this hypothesis, we conducted a comprehensive investigation with three sequential objectives: Firstly, to quantitatively assess the pollutant removal efficacy (NH_4_^+^-N, TN, TP, COD) of a commercial composite microbial inoculant under different operational modes, identifying the optimal “aerobic (aeration)-anoxic (external carbon source addition)” combination through laboratory-scale batch experiments. Secondly, to unravel the temporal dynamics of the bacterial community structure, identify the key functional genera driving nitrogen transformation, and elucidate the primary removal mechanisms under the optimal conditions. Thirdly, and most critically, to innovatively design, construct, and field-test a simple, partitioned in situ treatment system based on the above mechanistic insights and optimal parameters, evaluating its practical performance and stability over an extended period in a genuine black-odorous river channel.

## 2. Materials and Methods

### 2.1. Microbial Inoculum and Experimental Water

In this study, a representative multi-genus microbial consortium (comprising heterotrophic nitrifiers, aerobic denitrifiers, and organic degraders) was evaluated as a model system. Rather than assessing nominal commercial claims, the central scientific hypothesis posits that engineered redox cycling (sequenced aerobic-anoxic phases) can selectively enrich active heterotrophic nitrifying-aerobic denitrifying (HN-AD) taxa (*Pseudomonas* spp.), thereby bridging the gap between laboratory microbial succession and sustained field-scale nitrogen clearance. Prior to use, the powdered inoculum was revived according to the manufacturer’s instructions: 5 g of powder was dissolved in 1 L of sterile deionized water supplemented with 1 g of glucose and 0.1 g of sodium chloride. The mixture was shaken at 150 rpm and 30 ± 1 °C for 24 h to obtain the activated bacterial suspension for subsequent experiments [21].

The experimental water for the laboratory-scale study was synthetic wastewater simulating typical polluted river water. The primary components per liter were: 100.0 mg CH_3_COONa (as carbon source), 20.0 mg (NH_4_)_2_SO_4_ (as nitrogen source, equating to 4.24 mg/L NH_4_^+^-N), 5.0 mg KH_2_PO_4_ (as phosphorus source, equating to 1.14 mg/L PO_4_^3−^-P), 10.0 mg MgSO_4_·7H_2_O, 5.0 mg CaCl_2_, and 1.0 mL of a trace element solution. The trace element solution contained (per liter): 1.5 g FeCl_3_·6H_2_O, 0.15 g H_3_BO_3_, 0.03 g CuSO_4_·5H_2_O, 0.18 g KI, 0.12 g MnCl_2_·4H_2_O, 0.06 g Na_2_MoO_4_·2H_2_O, 0.12 g ZnSO_4_·7H_2_O, and 0.15 g CoCl_2_·6H_2_O. The initial pH of the synthetic wastewater was adjusted with NaHCO_3_ to 7.2–7.5.

### 2.2. Experimental Design and Operation

Laboratory-scale experiments were conducted to evaluate the pollutant removal efficacy of the composite microbial inoculant under different environmental conditions. Batch experiments were performed in cylindrical plexiglass reactors, each with a working volume of 2.0 L, maintained at room temperature (25 ± 2 °C) for a total duration of 27 days. A synthetic wastewater, formulated to simulate the characteristics of nitrogen-polluted receiving water, served as the experimental medium. To systematically investigate the effects of aeration and bioaugmentation, four distinct experimental groups were established in triplicate: (1) a Control Group (CG) containing only synthetic wastewater; (2) an Inoculum-only Group (Inoc) with synthetic wastewater amended with 20 mL of the activated bacterial suspension (equivalent to a 1% *v*/*v* inoculation ratio); (3) an Aeration-only Group (Aer) with synthetic wastewater under continuous aeration supplied via an air pump and fine-bubble diffusers to maintain dissolved oxygen (DO) levels above 3.0 mg/L; and (4) an Aeration + Inoculum Group (Aer + Inoc), which combined synthetic wastewater, the activated inoculum, and continuous aeration. This group was subjected to a defined operational strategy to promote sequential nitrification and denitrification. Specifically, on days 7, 14, and 21, sodium acetate was added as an external carbon source to provide a requisite electron donor for denitrification, immediately after which aeration was temporarily suspended for 24 h periods to create transient anoxic conditions (DO < 0.5 mg/L). Water samples (approximately 50 mL) were aseptically collected from each reactor at predetermined intervals (days 0, 1, 3, 7, 14, 21, and 27) for subsequent analysis of physicochemical parameters and microbial community composition. The 24 h aeration breaks were introduced precisely on days 7, 14, and 21 because preliminary tracking indicated that nearly all available NH_4_^+^-N had been oxidized by these intervals, resulting in peak accumulations of NO_2_-N and NO_3_-N. Halting aeration at these nodes created the necessary anoxic environment (DO < 0.5 mg/L) to activate denitrifying pathways.

### 2.3. Analytical Methods

Water quality parameters were analyzed following standard methods [22,23,24,25,26,27]. Dissolved oxygen (DO) was monitored in situ using a portable multi-parameter water quality meter (HQ40d, Hach, Loveland, CO, USA). Ammonium nitrogen (NH_4_^+^-N) was determined by Nessler’s reagent spectrophotometry. Nitrate (NO_3_^−^-N) and nitrite (NO_2_^−^-N) were measured by ultraviolet spectrophotometric method and N-(1-naphthyl)-ethylenediamine dihydrochloride spectrophotometric method, respectively. Total nitrogen (TN) was analyzed using alkaline potassium persulfate digestion followed by UV spectrophotometry. Chemical oxygen demand (COD) was measured via the rapid digestion-spectrophotometric method. Total phosphorus (TP) was determined by ammonium molybdate spectrophotometry after persulfate digestion. The removal efficiency (R, %) for a pollutant was calculated as: R = [(C_0_ − C_e_)/C_0_] × 100%, where C_0_ and C_e_ are the initial and final concentrations, respectively.

### 2.4. Microbial Community Analysis

Microbial biomass for community analysis was collected by filtering 200 mL of water sample from the Aer + Inoc group at different time points (days 0, 1, 3, 7, 12, 15, 18, 21, 25, 27) through 0.22-μm pore-size polycarbonate membrane filters. Total genomic DNA was extracted from the filters using the FastDNA^®^ Spin Kit for Soil (MP Biomedicals, Santa Ana, CA, USA) following the manufacturer’s protocol. The quality and concentration of the extracted DNA were checked using a NanoDrop 2000 spectrophotometer (Thermo Fisher Scientific, Wilmington, DE, USA). The V3–V4 hypervariable region of the bacterial 16S rRNA gene was amplified with universal primers 515F (5′-GTGYCAGCMGCCGCGGTAA-3′) and 806R (5′-CCGGACTACHVGGGTWTCTA AT-3′) [28,29,30,31]. Sequencing was performed on an Illumina MiSeq platform (Shanghai Majorbio Bio-pharm Technology Co., Ltd., Shanghai, China) to generate 250 bp paired-end reads. The raw sequencing data were processed using QIIME2 (version 2022.8). Sequences were quality-filtered, denoised, merged, and clustered into amplicon sequence variants (ASVs) using the DADA2 plugin. Taxonomic assignment of representative ASV sequences was conducted against the database SILVA v138.1. Alpha diversity indices (Chao1, Shannon) and beta diversity analysis (Principal Coordinates Analysis, PCoA, based on Bray–Curtis dissimilarity) were performed. Differential abundance of taxa between groups was assessed using linear discriminant analysis effect size (LEfSe).

### 2.5. Pilot-Scale System Design and Field Operation

Based on the laboratory findings, a pilot-scale partitioned in situ treatment system was designed and deployed for two months in a black-odorous river channel (approx. 8 m wide, 1.2 m average depth, average flow velocity 0.05 m/s) in Guangzhou, China. To smooth the transition from the controlled synthetic wastewater matrix to the complex environmental river matrix, a 15-day in situ acclimation and immobilization phase was executed at start-up, allowing the inoculated consortium to adapt to natural river chemistry and establish resilient biofilms on the carrier media before opening the continuous flow. The system consisted of two sequential functional zones built along a 20 m stretch of the riverbank: (1) An aerobic bio-contact oxidation zone: A 5 m (L) × 2 m (W) × 2 m (H) floating raft equipped with bundled elastic packing material (specific surface area > 250 m^2^/m^3^) and a solar-powered aeration device to maintain DO > 2.0 mg/L. (2) A hybrid anaerobic-anoxic zone: A 10 m (L) × 3 m (W) × 1.5 m (H) submerged baffle curtain area containing suspended microbial carrier media and slow-release carbon source blocks (composed of polycaprolactone and starch) to facilitate anoxic denitrification. Water from the upstream river flowed sequentially through these two zones. The same composite microbial inoculum was immobilized onto the media in both zones via a circulation and adsorption method at the system’s start-up. Influent (upstream river water) and effluent (from the anoxic zone) were sampled weekly. Water quality parameters (NH_4_^+^-N, NO_3_^−^-N, TN, COD, TP, DO, pH) were analyzed as described in Section 2.3.

### 2.6. Statistical Analysis

All laboratory experiments were performed in triplicate. Data are presented as mean ± standard deviation (SD). Differences in pollutant removal efficiencies and microbial alpha diversity indices among experimental groups and time points were evaluated using one-way analysis of variance (ANOVA) followed by Tukey’s honestly significant difference (HSD) post hoc test. Statistical significance was set at *p* < 0.05. All statistical analyses and graph plotting were conducted using SPSS software (version 26.0, IBM Corp., New York, NY, USA) and OriginPro 2022 (OriginLab Corp., Northampton, MA, USA), respectively.

## 3. Results

### 3.1. Pollutant Removal Performance of the Microbial Inoculant Under Different Conditions

**Figure 1:**
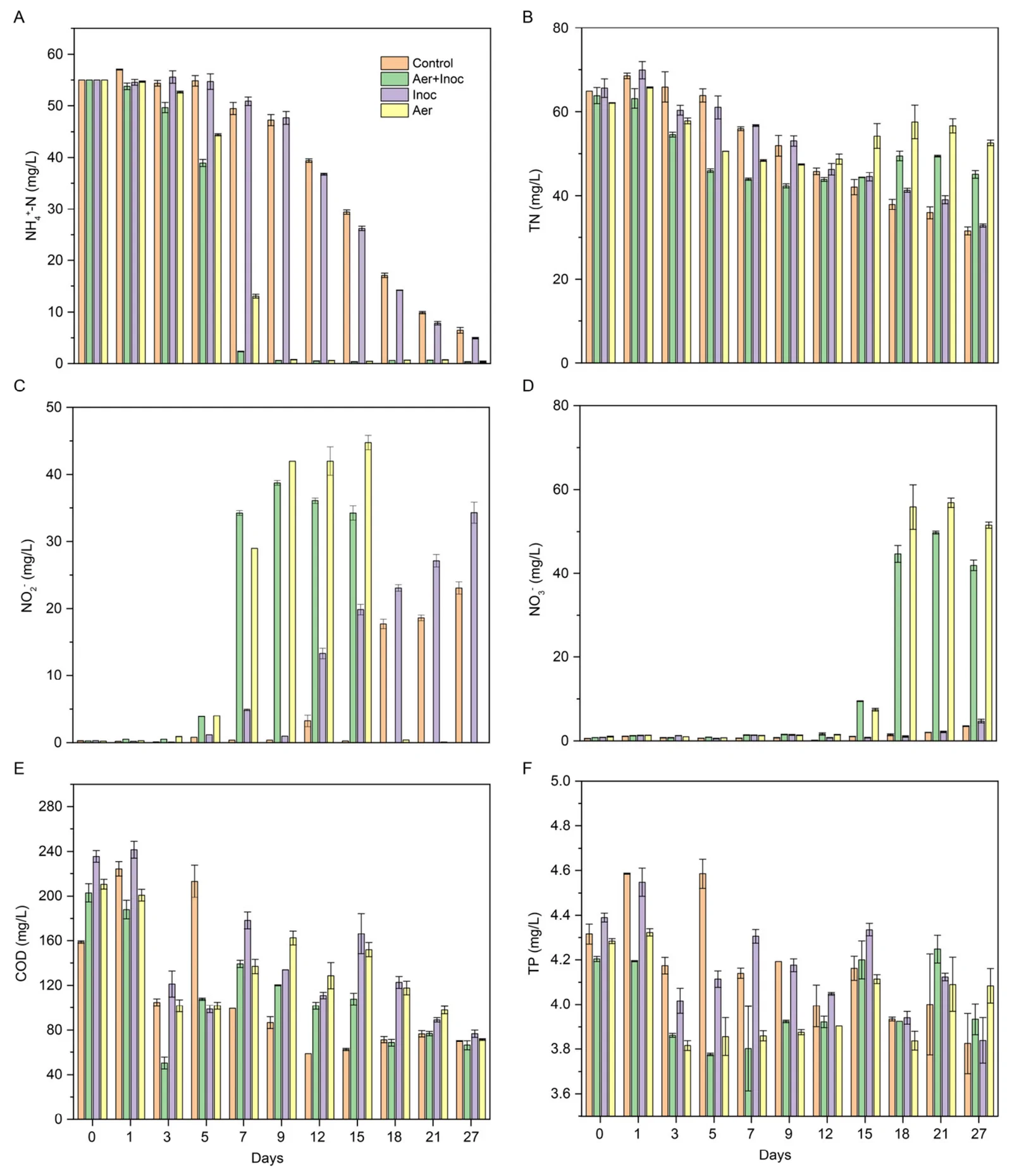
Temporal profiles of nitrogen species (NH_4_^+^-N, NO_2_^−^-N, NO_3_^−^-N, TN, COD, TP) in the Aeration + Inoculum (Aer + Inoc) group during the 27-day remediation process: (**A**) Ammonium nitrogen (NH_4_^+^-N) concentrations; (**B**) Total nitrogen (TN) concentrations; (**C**) Nitrite nitrogen (NO_2_^−^-N) concentrations; (**D**) Nitrate nitrogen (NO_3_^−^-N) concentrations; (**E**) Chemical oxygen demand (COD) levels; (**F**) Total phosphorus (TP) concentrations. Data are presented as mean ± standard deviation.

The removal efficiencies of key pollutants (NH_4_^+^-N, TN, NO_2_^−^-N, NO_3_^−^-N, COD, TP) under four experimental setups over 27 days (Figure 1). The control (CG) and inoculum-only (Inoc) groups showed limited and incomplete removal of NH_4_^+^-N, with concentrations remaining above 6.43 mg/L and 4.93 mg/L respectively by day 27 (Figure 1A). The aeration-only (Aer) group achieved significant NH_4_^+^-N removal (99.4%). However, the most rapid assimilation kinetics were observed in the aeration + inoculum (Aer + Inoc) group, where the NH4^+^-N concentration plummeted from the initial baseline to 2.4 mg/L by Day 7, achieving a rapid initial removal efficiency of 95.6% (Figure 1A). Crucially, the systematic tracking of nitrification intermediates explained the severe performance bottleneck observed in the Aer group. Although the Aer group effectively oxidized ammonium, its overall TN removal efficiency was restricted to only 15.3%. (Figure 1B). This was primarily due to the massive accumulation of nitrification intermediates and products, In the Aer + Inoc group with NO_2_^−^-N concentration rapidly increased and peaked at 38.75 mg/L on day 9 (Figure 1C), before dropping to near-zero levels after day 18 and NO3^−^-N soared from an initial 0.75 mg/L to 41.96 mg/L by the end of the experiment (day 27) (Figure 1C). Conversely, the highest and most comprehensive nutrient clearance was successfully achieved in the Aer + Inoc group. By successfully coupling heterotrophic nitrification with active denitrification during the structured aeration breaks, the transiently accumulated NO_2_^−^-N and NO_3_^−^-N in the Aer + Inoc group were systematically eliminated after Day 18 and Day 21, respectively. This resulted in a remarkable terminal TN concentration of 2.6 mg/L by Day 27, translating to a superior total nitrogen removal efficiency of 95.90% (Figure 1D). Additionally, the Aer + Inoc group excelled in concurrent organic matter and phosphorus treatment, achieving COD and TP removal efficiencies of 67.4% and 6.4%, respectively, while maintaining the most stable and consistent decreasing trends for COD throughout the operation (Figure 1E,F).

The temporal variation of nitrogen species in the Aer + Inoc group under continuous aeration is detailed in Figure 1. Following inoculation, NH_4_^+^-N was rapidly oxidized, yet the lack of anoxic phases led to significant NO_x_^−^-N accumulation. NO_2_^−^-N peaked at 38.75 mg/L on day 9, while NO_3_^−^-N soared to 41.96 mg/L by day 27. Consequently, despite efficient nitrification, the TN removal efficiency remained limited at 15.3% (Figure 1D), highlighting the necessity of introducing intermittent anoxic phases for complete denitrification.

### 3.2. Microbial Community Dynamics and Succession

**Figure 2:**
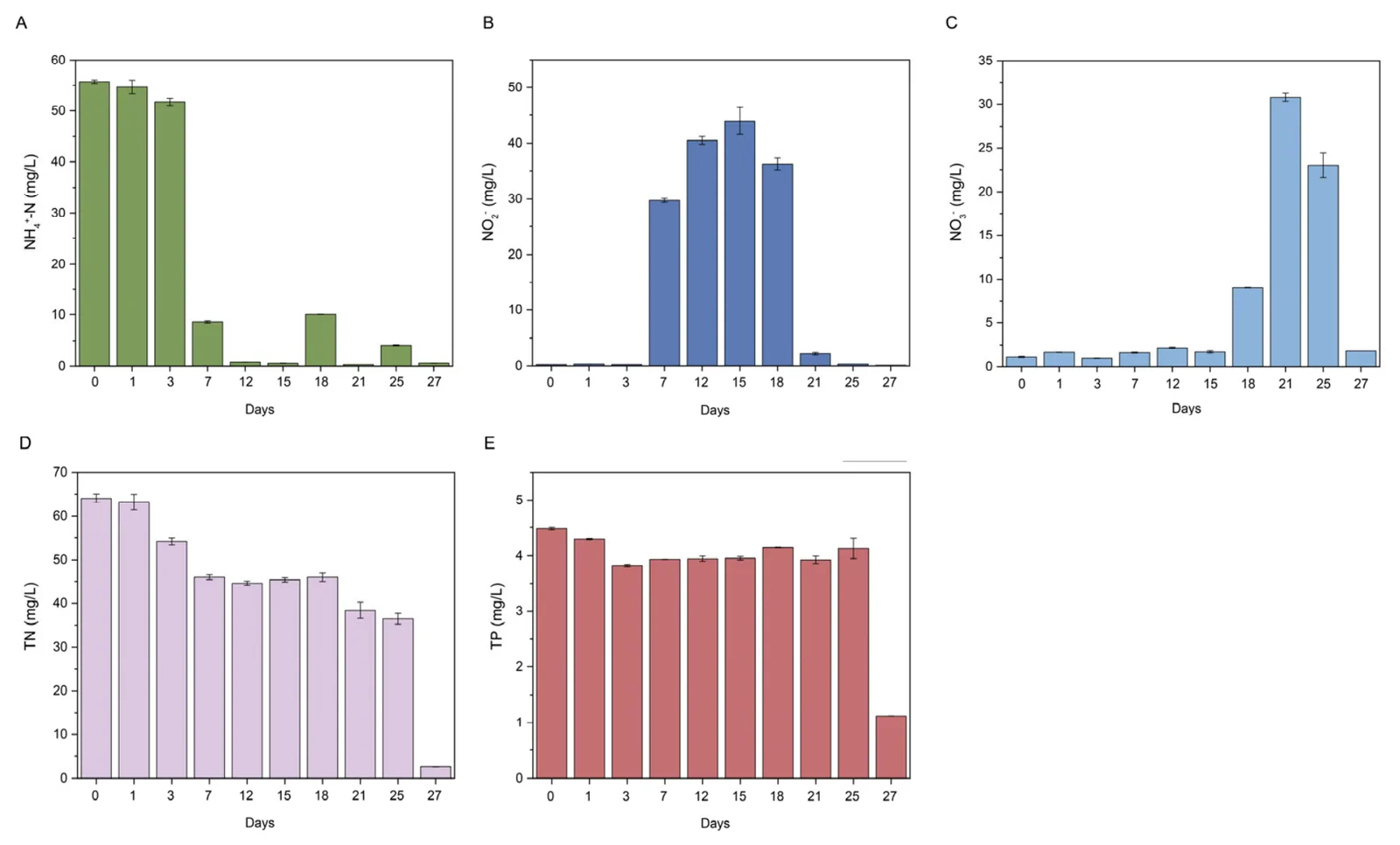
Temporal variations of nitrogen species and total phosphorus in the Aer + Inoc treatment group during the 27-day remediation process: (**A**) Ammonium nitrogen (NH_4_^+^-N) concentrations; (**B**) Nitrite nitrogen (NO_2_^−^-N) concentrations; (**C**) Nitrate nitrogen (NO_3_^−^-N) concentrations; (**D**) Total nitrogen (TN) concentratio; (**E**) Total phosphorus (TP) concentrations.

To resolve the denitrification suppression observed under continuous aeration (Figure 1), the 27-day sequential control experiment introduced periodic redox transitions (Figure 2). The temporal evolution of pollutants in the Aer + Inoc group exhibited a distinct three-stage nitrogen transformation (Figure 2). During the first 15 days, NH_4_^+^-N was rapidly oxidized from 55.6 to <1.0 mg/L (Figure 2A), triggering a significant accumulation of NO_2_^−^-N which peaked at 44.1 mg/L (Day 15), confirming that nitritation was the primary metabolic pathway during the initial phase. In Stage II (Days 15–21), NH_4_^+^-N remained strictly constrained at near-zero levels (<0.5 mg/L), while NO_2_^−^-N rapidly decreased to 12.4 mg/L (Figure 2B). Subsequently, a transition from nitritation to nitratation occurred between days 18 and 21, with NO_3_^−^-N soaring to 30.8 mg/L (Figure 2C). A critical breakthrough in nutrient removal was observed in the final stage (Days 21–27). Both NO_2_^−^-N and NO_3_^−^-N were nearly exhausted, leading to a terminal TN concentration of 2.6 mg/L (Figure 2D). Concurrently, TP concentrations—which remained recalcitrant at ~4.0 mg/L for the majority of the experiment—sharply declined to 1.1 mg/L by day 27 (Figure 2E), this synchronization with the complete nitrogen clearance demonstrates that the late-stage anoxic settings coupled with carbon availability facilitated concurrent denitrification and biological phosphorus immobilization.

**Figure 3:**
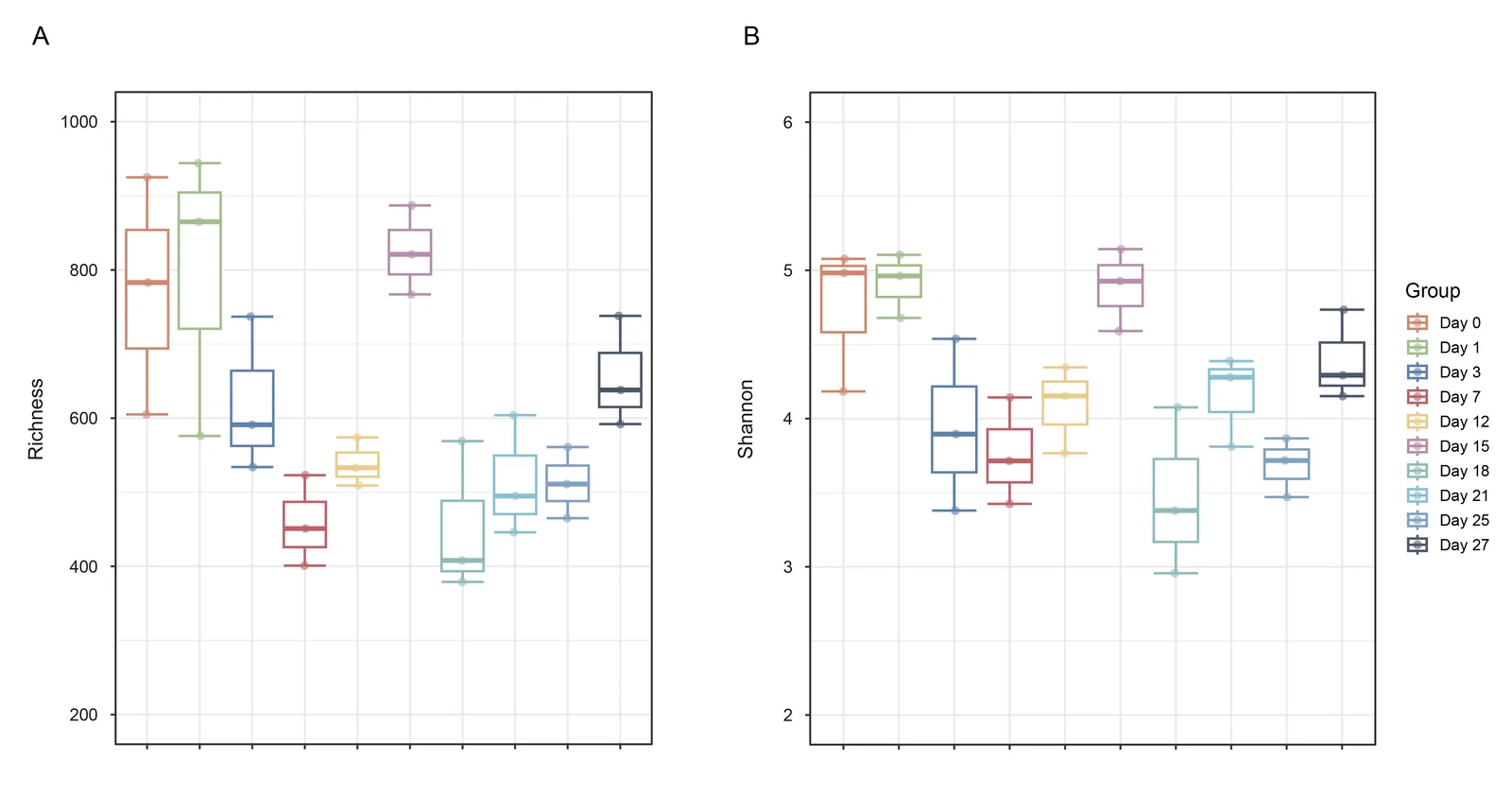
Dynamics of bacterial alpha diversity in the Aer + Inoc group throughout the 27-day remediation period:(**A**) Richness index; (**B**) Shannon index.

High-throughput sequencing of the 16S rRNA gene was performed on samples from the Aer + Inoc group across ten time points (from Day 0 to Day 27) to elucidate microbial community shifts. Alpha diversity indices (Richness and Shannon) Initially, both indices showed a sharp decline from Day 1 to Day 7, where the Richness and Shannon values reached their first local minima. This reduction suggests a strong environmental filtering effect in the early stage of treatment, leading to the selection of a more specialized microbial community (Figure 3).

**Figure 4:**
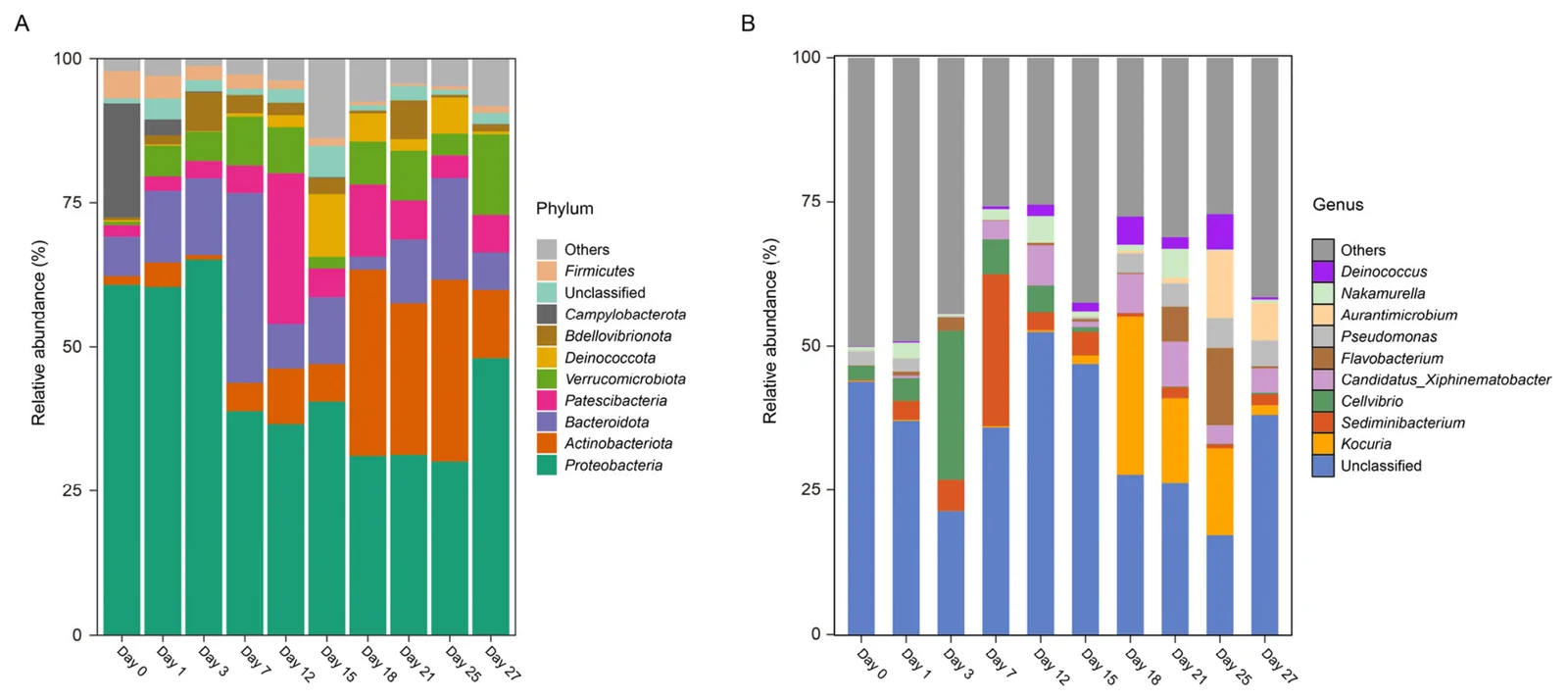
Microbial community composition at (**A**) phylum and (**B**) genus level in the Aer + Inoc group at different time points (Day 0, 7, 14, 27). Only top 10 abundant phyla/genera are shown.

At the phylum level (Figure 4A), the microbial community was predominantly composed of *Proteobacteria*, *Actinobacteriota*, and *Bacteroidota*. Initially, *Proteobacteria* was the most abundant phylum (Day 0–3); however, its relative abundance underwent a noticeable decline starting from Day 7, coinciding with the proliferation of other groups. The relative abundance of major genera shifted dramatically (Figure 4B). In the early stage, *Sediminibacterium* (a member of *Bacteroidota*) increased markedly, peaking at Day 7. From Day 15 to Day 25, the genus *Kocuria* (belonging to *Actinobacteriota*) became highly prominent, reaching its maximum relative abundance during this period. Other functional genera, such as *Cellvibrio* and *Candidatus*_Xiphinematobacter, also exhibited temporal fluctuations. Other genera known for denitrification capabilities, such as *Pseudomonas*, *Flavobacterium*, and *Arenimonas*, also showed increased relative abundance as the experiment progressed. Notably, typical autotrophic ammonia-oxidizing bacteria (AOB, e.g., *Nitrosomonas*) and nitrite-oxidizing bacteria (NOB, e.g., *Nitrobacter*) were not detected among the dominant genera at the sequencing depth used.

**Figure 5:**
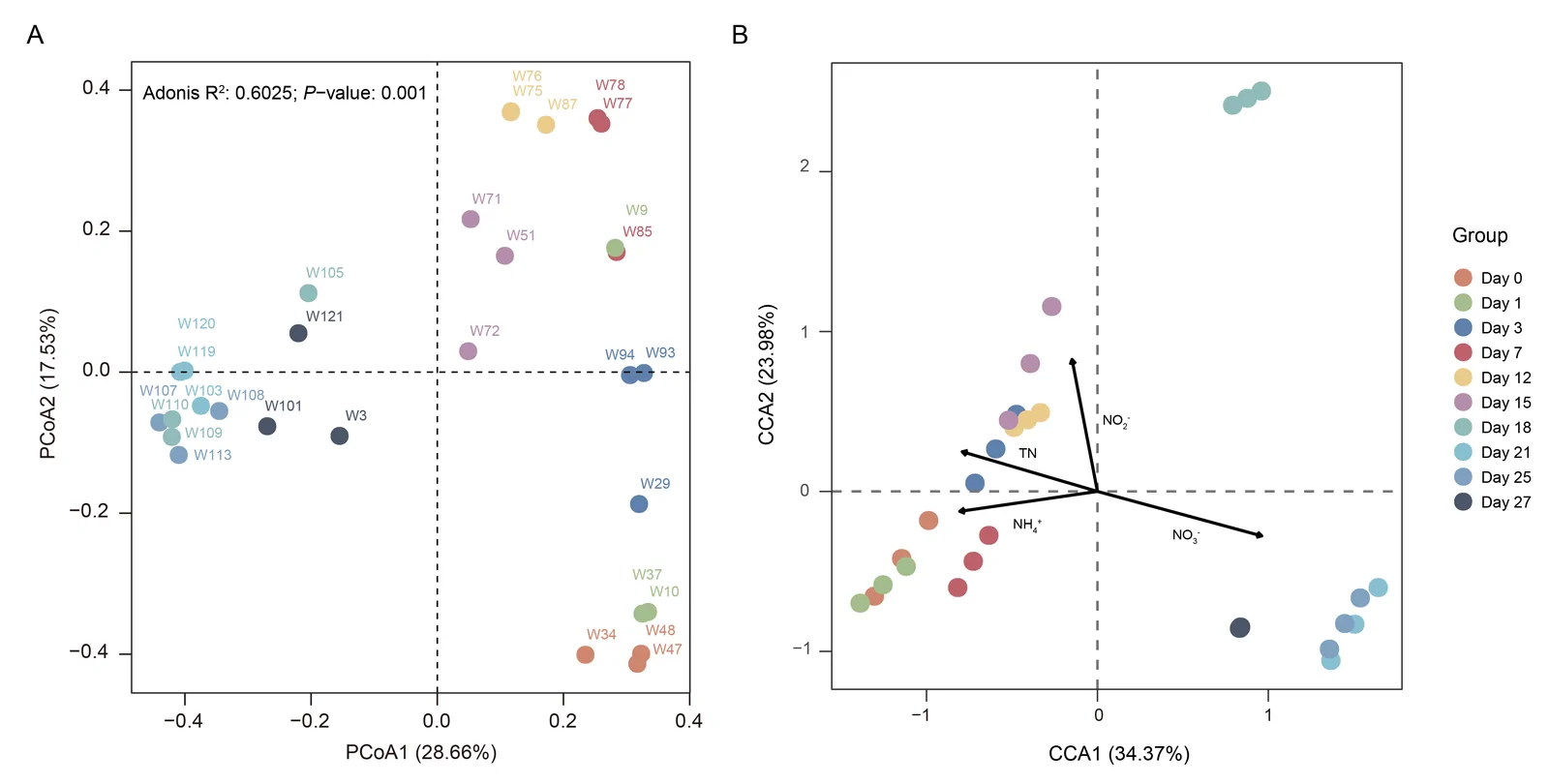
Multivariate analysis of bacterial community succession and its correlation with environmental factors in the Aer + Inoc group:(**A**) Principal Coordinate Analysis (PCoA) plot showing the temporal dynamics of bacterial community structure; (**B**) Canonical Correspondence Analysis (CCA) plot illustrating the relationships between bacterial community composition and environmental factors (NH_4_^+^-N, NO_2_^−^-N, NO_3_^−^-N, TN).

PCoA (Figure 5A) revealed a significant temporal succession in microbial communities. Early-stage samples (Day 0–7) showed high variability, while late-stage samples (Day 18–27) migrated along PCoA1 to form a tighter cluster, indicating a transition from an unstable state to a specialized, stable configuration under selective pressure. CCA (Figure 5B) further demonstrated that this shift was driven by nitrogen dynamics: early communities correlated with NH_4_^+^-N and TN, whereas mid-to-late stages shifted toward NO_2_^−^-N and NO_3_^−^-N vectors. Overall, environmental filtering by nitrogen species successfully shaped a functional community optimized for wastewater treatment.

### 3.3. Performance of the Pilot-Scale Partitioned In Situ Treatment System

**Figure 6:**
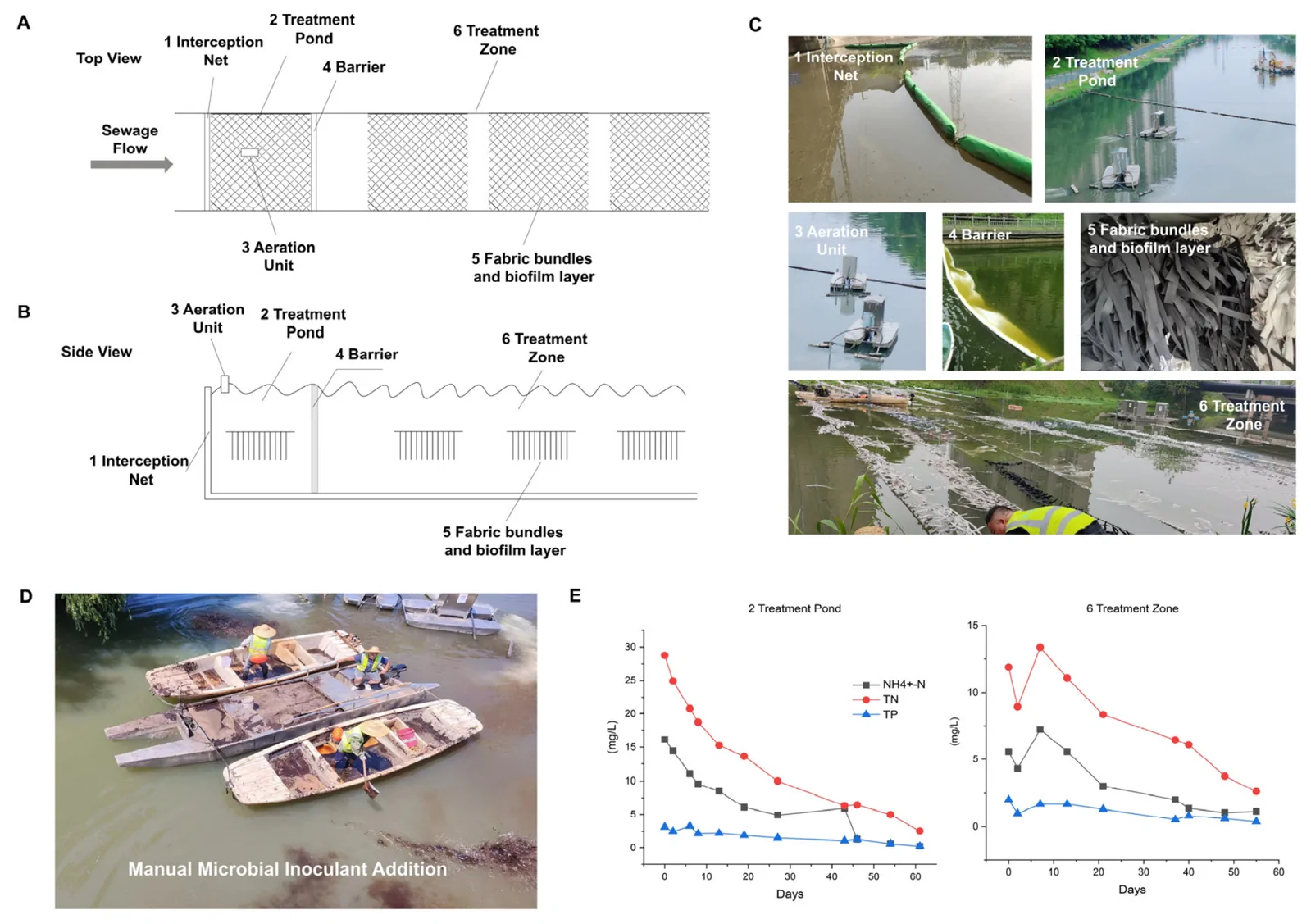
Performance of the pilot-scale partitioned system over 60 days of operation:(**A**) Top view schematic diagram of the system; (**B**) Side view schematic diagram of the system; (**C**) Photographs of key functional components in the field; (**D**) Field operations showing manual microbial inoculant addition; (**E**) Temporal variations of NH_4_^+^-N, TN, and TP concentrations in the treatment pond and treatment zone over 60 days.

Based on the lab-scale findings, a pilot-scale “Aerobic bio-contact oxidation + hybrid anaerobic-anoxic” partitioned system was constructed and operated for 60 days in a black-odorous river channel (Figure 6). The system effectively improved water quality. As shown in Figure 6E, the concentration of NH_4_^+^-N in the influent river water fluctuated between 1.1 mg/L and 16.2 mg/L. The system effluent maintained stable and low NH_4_^+^-N levels, predominantly below 1.2 mg/L after the initial 15-day acclimation period ). The average NH_4_^+^-N removal rate was 91.7%. The system showed robust nitrogen removal capabilities. The initial TN concentration in the influent was as high as 28.8 mg/L. Following the treatment process, the effluent TN was reduced significantly to 2.5 mg/L in the Treatment Pond and 2.6 mg/L in the Treatment Zone. The TP levels were maintained at a relatively low range throughout the process. The influent TP (approx. 2.0–3.2 mg/L) was gradually reduced to below 0.4 mg/L by the end of the experiment, indicating effective phosphorus immobilization or biological uptake. Evaluating overall removal dynamics, the TN removal efficiency plunged upward post-acclimation, stabilizing at an average of 91.3% in the Treatment Pond, while TP removal efficiency exhibited a steady, progressive increase, advancing from early fluctuations to a terminal peak approaching 90%by Day 60. This synchronized enhancement confirms that the partitioned framework successfully translated laboratory-scale HN-AD and phosphorus immobilization kinetics into long-term field stability. Overall, the system achieved average removal efficiencies of approximately 93.2% for NH_4_^+^-N and 91.3% for TN (based on Treatment Pond data). The water appearance changed from turbid to noticeably clearer, with a significant reduction in foul odors, confirming the successful ecological restoration of the river water.

## 4. Discussion

### 4.1. Integrated Evidence from Lab to Field Validates the Engineered Bioaugmentation Approach

This study presents a comprehensive investigation that bridges the gap between laboratory-scale bioaugmentation potential and field-scale application for nitrogen removal in receiving waters. The laboratory experiments unequivocally demonstrated that the synergistic combination of aeration (providing aerobic conditions for nitrification) and the composite inoculum, supplemented with strategic anoxic phases induced by external carbon addition, was indispensable for achieving high and simultaneous removal of NH_4_^+^-N (95.60%) and TN (95.90%). This performance far surpassed that of aeration alone or inoculum addition alone, highlighting the critical role of bioaugmentation in enabling complete denitrification, a process often limited by the lack of suitable indigenous denitrifiers or carbon sources in many aquatic environments. Microbial community analysis further confirmed the mechanistic link, revealing a dynamic succession towards a community dominated by known heterotrophic denitrifiers (e.g., *Sediminibacterium* and *Kocuria*) following anoxic phases, which correlated directly with the observed TN removal. Most significantly, the translation of these mechanistic insights into a simple, partitioned pilot-scale system and its successful operation for two months in a black-odorous river channel, achieving >91% removal of both NH_4_^+^-N and TN, provides strong empirical validation for the feasibility of this integrated bioaugmentation-engineering approach.

### 4.2. Engineered Redox Conditions Unlock the Synergistic Potential of the Composite Inoculant

The superior performance of the “Aeration + Inoculum” (Aer + Inoc) group underscores a fundamental principle in bioaugmentation for complex nitrogen removal: the introduced consortium must be compatible with and thrive under the engineered environmental conditions designed to select for the desired metabolic functions [32,33,34]. While aeration alone effectively promoted nitrification, leading to a peak of NO_2_^−^-N (38.75 mg/L), it failed to achieve substantial TN removal due to the lack of an active denitrifying community and/or insufficient bioavailable carbon. The addition of the composite inoculum under the same aeration regime did not initially improve TN removal until an external carbon source was introduced and aeration was temporarily halted. This indicates that the inoculum contained a reservoir of denitrification-capable bacteria whose activity was contingent upon the creation of anoxic conditions and the availability of an easily degradable carbon source like sodium acetate. The intermittent anoxic phase strategy proved highly effective, triggering rapid reduction of accumulated NO_3_^−^-N and NO_2_^−^-N without compromising the subsequent recovery of nitrification activity once aeration resumed. This cyclic “feast-famine” strategy for denitrifiers, driven by controlled carbon and oxygen availability, is a recognized method for enhancing nitrogen removal efficiency in engineered systems [35,36].

Our findings align with and extend recent work on optimizing bioaugmentation strategies. For instance, a study on enhancing biofilm processes for wastewater treatment through bioaugmentation with *Methylobacterium gregans* demonstrated that the success of inoculant integration heavily depends on creating niche conditions favorable for the augmented strain [10]. Similarly, research on artificial microbial consortia engineering for landfill leachate treatment emphasized the importance of designing operational conditions (like C/N ratio and redox potential) that promote the synergistic interactions within the consortium [12]. Our study contributes to this body of knowledge by explicitly demonstrating this principle for a commercial composite inoculant in a simulated river water context and by quantitatively linking specific operational modes (continuous aeration vs. intermittent anoxia) to process performance.

### 4.3. Microbial Community Succession Pinpoints the Functional Drivers of Nitrogen Removal

The 16S rRNA gene sequencing data provided crucial insights into the microbial ecology underpinning the observed treatment performance. The significant decrease in alpha diversity (Richness and Shannon index) exhibited a fluctuating “V-shaped” pattern, with a sharp decrease by Day 7 followed by a significant transient recovery on Day 15 [18,19]. The overwhelming dominance of *Proteobacteria* throughout the experiment is consistent with its well-known role as a key phylum in wastewater treatment and nitrogen cycling ecosystems [5,37]. The most striking observation was the temporal succession of dominant genera and its correlation with process phases. The genus *Sediminibacterium* (*Bacteroidota*) increased markedly during the initial phase (Day 7), while *Kocuria* (*Actinobacteriota*) became highly prominent during the mid-to-late stages (Day 15–25), corresponding to the peak of nitrite oxidation and nitrate production. The low relative abundance or absence of typical autotrophic nitrifiers (AOB/NOB) among the dominant ASVs suggests that nitrification in this system might have been carried out by less canonical or heterotrophic nitrifiers present within the *Proteobacteria* and other phyla, or that their abundance was below the detection threshold of our sequencing approach but their activity was significant. This aligns with growing recognition of the diversity and ecological importance of non-canonical nitrifiers in various environments [17,38,39]. Notably, the substantial enrichment of *Kocuria* (*Actinobacteriota*) and *Sediminibacterium* (*Bacteroidota*) during and after the treatment phases points to their potential role in the observed nitrogen transformation. Their metabolic versatility, including the ability to utilize various carbon sources under low-oxygen conditions, likely made them highly competitive in our system following acetate addition. The co-enrichment of other known denitrifying genera like *Pseudomonas* and *Flavobacterium* further supports the establishment of a robust denitrifying guild responsible for the observed TN removal.

This community shift towards denitrifiers in response to anoxic/carbon-rich conditions is a direct microbial echo of the process performance data. It exemplifies the concept of “ecological engineering” of microbial communities through manipulation of environmental parameters [40,41]. A recent 2024 study on microbial community dynamics during process upgrading in a full-scale plant also observed similar rapid and functional restructuring of communities in response to operational changes [42,43]. Our results reinforce that successful bioaugmentation is not merely about adding bacteria but about managing the ecosystem to select for and sustain the desired functional groups from the augmented inoculum.

### 4.4. Translating Mechanistic Insights into a Functional Pilot-Scale Treatment System

The design of the pilot-scale “Aerobic Bio-contact Oxidation + Hybrid Anaerobic-Anoxic” system was a direct translation of the laboratory-identified optimal conditions and mechanistic understanding. The aerobic zone, equipped with high-surface-area media and solar-powered aeration, was intended to replicate the continuous aeration condition that supported nitrification. The subsequent hybrid anoxic zone, incorporating slow-release carbon source blocks, was designed to provide the necessary anoxic environment and sustained carbon supply for denitrification, mirroring the intermittent anoxic phases created in the lab. This physical separation of redox zones is a classic and effective strategy in wastewater treatment (e.g., A/O, A^2^/O processes) to prevent competition between aerobic and anoxic processes and to allow for independent optimization of each stage [4,20].

The successful field performance (average NH_4_^+^-N and TN removal > 91%) validates this design logic for in situ river water treatment. The system effectively transformed a stretch of flowing water into a sequential bioreactor. The use of attached-growth media (in the aerobic zone) and suspended carriers (in the anoxic zone) provided protected niches for microbial colonization and retention, which is critical for maintaining the activity of the augmented inoculum in flowing water, where washout is a major challenge [44,45]. The slow-release carbon source addressed the common limitation of insufficient carbon for denitrification in many receiving waters, which often have low C/N ratios [46,47].

Comparing our system to other recent in situ bioaugmentation efforts provides context. Jiao et al. (2024) reported successful bioaugmentation of a biological contact oxidation ditch with indigenous nitrifying bacteria for river water remediation, achieving significant NH_4_^+^-N removal but with less emphasis on TN control [11]. Another 2024 study on an integrated anaerobic/anoxic/aerobic-membrane aerated biofilm reactor (AOA-MABR) highlighted the effectiveness of combining multiple redox zones for advanced nitrogen removal, though in a more complex and energy-intensive reactor configuration [3]. Our pilot system demonstrates that a relatively simple, solar-assisted, partitioned design based on fundamental nitrification-denitrification principles can achieve comparable high-level nitrogen removal, offering a potentially more cost-effective and manageable solution for decentralized or remote water bodies.

### 4.5. Implications, Limitations, and Future Perspectives

This study has several important implications. Firstly, it provides a validated framework for developing in situ bioaugmentation technologies: starting with targeted laboratory evaluation under simulated redox conditions, elucidating the microbial mechanisms, and then using these insights to inform the design of field-applicable engineering systems. Secondly, it highlights that the functional success of a commercial composite inoculum is highly dependent on the engineered environment created for it. The inoculum served as a “seed bank” of diverse genetic potential, but its expression into desired ecosystem functions (complete nitrification-denitrification) was unlocked only by the appropriate “aerobic-anoxic” operational strategy.

However, several limitations should be acknowledged. First, the metagenomic analysis was limited to 16S rRNA gene sequencing. While this revealed community structure shifts, it does not provide direct evidence of functional gene abundance (e.g., *amoA*, *nirK*, *nirS*, *nosZ*) or activity.

Future studies employing metatranscriptomics or qPCR for key functional genes would strengthen the mechanistic claims by directly linking taxonomic changes to nitrogen-cycling potentials [48]. Second, the pilot study was conducted over two months during a specific season. The long-term stability of the system across seasonal variations in temperature, rainfall, and pollutant loading remains to be evaluated. Factors such as biofilm aging, carrier clogging, and the depletion/regeneration of the slow-release carbon source need monitoring over extended periods [49]. Third, continuous pH logging was not conducted during the lab-scale batch experiments. Although the synthetic wastewater was buffered with KH_2_PO_4_ and NaHCO_3_ (initial pH 7.2–7.5), and the high nitrogen removal efficiency (95.9%) indicates that pH remained within a biologically favorable range, the lack of real-time pH data limits our ability to correlate microbial metabolic shifts with proton flux dynamics. Future studies will integrate high-frequency pH probes to quantify these interactions. Fourth, the economic feasibility and life-cycle environmental impact of this approach, including the cost of inoculum production, carrier media, and solar panel maintenance, require a comprehensive assessment for broader application [50,51], Future research should focus on: (1) optimizing the operational parameters of the partitioned system, such as the hydraulic retention time (HRT) in each zone, the aeration intensity/carbon release rate ratio, and strategies for dealing with fluctuating inflows; (2) investigating the potential for using waste-derived or cheaper alternative carbon sources (e.g., agricultural by-products) in the anoxic zone to improve sustainability; (3) exploring the integration of this bioaugmentation-partitioned process with other technologies, such as phytoremediation or adsorption materials, for the concurrent removal of other pollutants like phosphorus and heavy metals; (4) conducting life-cycle and techno-economic analyses to compare this approach with other in situ remediation technologies.

## 5. Conclusions

This study demonstrates that the composite microbial inoculant achieves optimal and synergistic nitrogen removal within a mechanistically structured aerobic-anoxic operational framework. Laboratory results confirmed that the cyclic provision of aerobic conditions (for nitrification) followed by anoxic phases induced by external carbon addition (for denitrification) is essential, enabling near-complete removal of both NH_4_^+^-N (95.6%) and TN (reaching a terminal concentration of 2.6 mg/L), robust organic degradation (67.4% COD removal) over 27 days. Microbial community analysis revealed that this performance was underpinned by a functional succession, with a significant enrichment of denitrifying taxa, particularly the genus *Kocuria* and *Sediminibacterium*, following the anoxic phases. Critically, these mechanistic insights were successfully translated into the rational design and implementation of a pilot-scale, partitioned in situ treatment system. Its open-field operation over two months in a black-odorous river channel successfully validated the practical feasibility of the spatial partition design, sustaining average field-scale removal efficiencies of 93.2% for NH_4_^+^-N and 91.3% for TN. Collectively, this work provides a validated model that explicitly links the understanding of microbial community dynamics under designed redox conditions to the development of effective, field-applicable bioaugmentation strategies, offering a promising and manageable approach for the remediation of nitrogen-polluted receiving waters.

## Data Availability

Data analyzed in this study are available in the NCBI BioProject database under accession number PRJNA1458030 (https://www.ncbi.nlm.nih.gov/bioproject/?term=PRJNA1458030 (accessed on 5 August 2026)).
